# Interdependence between nanoclusters AuAg_24_ and Au_2_Ag_41_

**DOI:** 10.1038/s41467-021-21131-5

**Published:** 2021-02-03

**Authors:** Danyu Liu, Wenjun Du, Shuang Chen, Xi Kang, Along Chen, Yaru Zhen, Shan Jin, Daqiao Hu, Shuxin Wang, Manzhou Zhu

**Affiliations:** 1 Department of Chemistry and Centre for Atomic Engineering of Advanced Materials, Anhui Province Key Laboratory of Chemistry for Inorganic/Organic Hybrid Functionalized Materials, Anhui University, Hefei, Anhui People’s Republic of China; 2 Key Laboratory of Structure and Functional Regulation of Hybrid Materials, Anhui University, Ministry of Education, Hefei, Anhui People’s Republic of China; 3 Institutes of Physical Science and Information Technology, Anhui University, Hefei, Anhui People’s Republic of China; 4 College of Materials Science and Engineering, Qingdao University of Science and Technology, Qingdao, People’s Republic of China

**Keywords:** Nanoparticles, Synthesis and processing

## Abstract

Whole series of nanoparticles have now been reported, but probing the competing or coexisting effects in their synthesis and growth remains challenging. Here, we report a bi-nanocluster system comprising two ultra-small, atomically precise nanoclusters, AuAg_24_(SR)_18_^−^ and Au_2_Ag_41_(SR)_26_(Dppm)_2_^+^ (SR = cyclohexyl mercaptan, Dppm = bis(diphenylphosphino)-methane). The mechanism by which these two nanoclusters coexist is elucidated, and found to entail formation of the unstable AuAg_24_(SR)_18_^−^, followed by its partial conversion to Au_2_Ag_41_(SR)_26_(Dppm)_2_^+^ in the presence of di-phosphorus ligands, and an interdependent bi-nanocluster system is established, wherein the two oppositely charged nanoclusters protect each other from decomposition. AuAg_24_(SR)_18_ and Au_2_Ag_41_(SR)_26_(Dppm)_2_ are fully characterized by single crystal X-ray diffraction (SC-XRD) analysis – it is found that their co-crystallization results in single crystals comprising equimolar amounts of each. The findings highlight the interdependent relationship between two individual nanoclusters, which paves the way for new perspectives on nanocluster formation and stability.

## Abstract (web summary)

Despite recent progress in individual nanocluster synthesis, understanding the competing or coexisting effects between particles in solution remains challenging. Here, the authors present the synthesis of a bi-nanocluster system comprising two atomically precise nanoclusters, and map out the interdependent relationship between them.

## Introduction

The synthesis and properties of individual nanoparticles are intriguing^1–5^ and have been widely studied^6–15^. In particular, the ligands used can profoundly affect the structure and composition of nanoparticles. For example, by using thiol ligands instead of R_4_N^+^, ultra-stable gold nanoparticles could be synthesized^16–20^. In addition, nanoparticle optical^21,22^, catalytic^23–25^, and magnetic^26,27^ properties can be controlled by modification of the structure, composition, and capping ligands of individual nanoparticles. In comparison, little is known about the interactions between nanoparticles, mainly due to their poly-disperse nature and the lack of effective in situ detection technologies. One recent breakthrough was the characterization of metal exchange behavior between two atomically precise nanoparticles^28–30^. Nonetheless, our understanding of the interactions between metal nanoparticles lags behind that of the individual nanoparticles themselves.

The methodology for exploring intermolecular interactions at the initial stage helps to regulate the synthesis of nanoparticles^31–34^. For example, the size focusing method for synthesizing atomically precise nanoparticles (nanoclusters), wherein the influence of reaction conditions (reaction temperature, growth kinetics, ligand bulkiness, etching time, ligand/metal ratio) on product nanoparticle size distribution are mapped out and then adjusted such that a specific size of product nanoparticles are obtained, is quite similar to the concept of “survival of the fittest”^35,36^. High-yield syntheses of molecularly pure Au_38_(SR)_24_^37^, Au_144_(SR)_60_^38^_,_ Au_64_(SR)_32_^39^ have all been accomplished with this size-focusing methodology. However, the size focusing method does not necessarily result in monodisperse nanoclusters, and is more likely to afford a mixture of several different monodisperse nanoclusters. These nanoclusters can be regarded as the “fittest” in the environment in which they were formed, but although they can be separated and fully characterized, such studies generally do not inform our understanding of the relationship between them. Important open questions include: when in solution, how do nanoparticles interact with each other, leading to the coexistence of two or more nanoparticles, which are mutually beneficial? Can nanoparticles exist in the presence of each other, but not separately? The discovery of such phenomena will lead us to a deeper understanding of the formation and growth mechanism of nanoparticles, and will provide effective guideline for the design of new nanoparticles, especially metal nanoparticles with precise structure.

Herein, we report the synthesis and characterization of a bi-nanocluster system, AuAg_24_ and Au_2_Ag_41_, and the interdependent relationship between these two nanoclusters are mapped out. By working with precisely structured nanoclusters, we obviated the problem of poly-dispersion, which plagues the study of nanoparticles. The relatively simple nature of this bi-nanocluster system also ensures that the exterior synthetic environment is the same for each individual nanoparticle, and therefore the interactions between them are more tractable. For example, we were able to determine that the negatively charged nanocluster AuAg_24_(SR)_18_^−^ (denoted as AuAg_24_) is produced first, and then some of it is converted into a positively charged nanocluster Au_2_Ag_41_(SR)_26_(Dppm)_2_^+^ (denoted as Au_2_Ag_41_). The two types of nanoclusters protect each other from decomposition, revealing the interdependent relationship between them. Moreover, co-crystallization of AuAg_24_ and Au_2_Ag_41_ resulted in crystals consisting of each of them in a molar ratio of 1:1.

## Results

### Synthesis and characterization

**Fig. 1:**
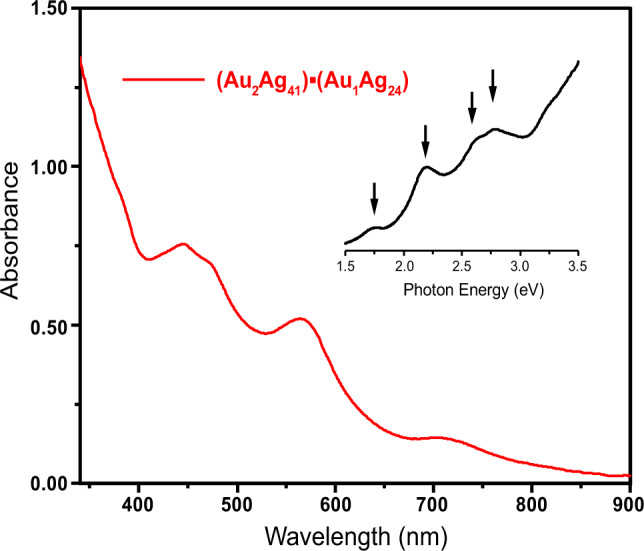
UV−vis absorption spectrum of the (Au_2_Ag_41_)■(AuAg_24_) co-crystal dissolved in CH_2_Cl_2_. The insert shows the UV–Vis spectrum plotted on the photon energy scale.

The synthesis of the title nanoclusters was performed in a one-pot method. First, the stable precursor Ag-Au-Dppm complex was synthesized as a solution in toluene. Introduction of the thiol ligand in the presence of NaBH_4_ resulted in the gradual formation of the nanoclusters. By adding 5 mL of n-hexane to the saturated toluene solution, single crystals could be obtained after 7 days. The resulting black single crystal was subjected to X-ray single crystal structure analysis (Supplementary Fig. 1), which revealed the co-crystallization of both AuAg_24_ and Au_2_Ag_41_ in a 1:1 molar ratio, and their hierarchical assembly in the triclinic space group. UV–Vis absorption spectrum of the (Au_2_Ag_41_)■(AuAg_24_) co-crystal showed intense peaks at ~443, 472, and 570 nm and a weaker peak at ~710 nm (Fig. 1), corresponding to excitation energies of 2.80, 2.63, 2.17, and 1.75 eV, respectively.

### Atomic structure

**Fig. 2:**
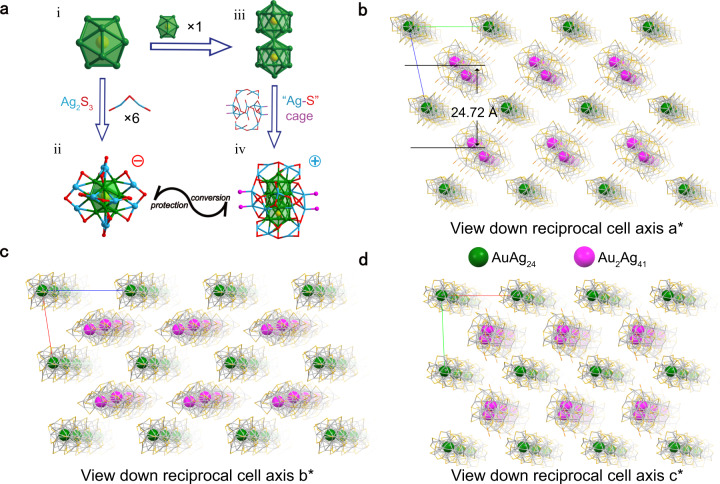
X-ray structure and packing model of (Au_2_Ag_41_)■(AuAg_24_) co-crystal. **a** Detail analysis of AuAg_24_ and Au_2_Ag_41_ nanocluster. Color labels: yellow, Au; green or sky blue, Ag; red, S; fuchsia, P. All C and H atoms are omitted for clarity; **b**, **c**, and **d** view down reciprocal cell axis a*, b*, and c*, respectively. The interlayer distance is 24.72 Å. The packing mode of (Au_2_Ag_41_)■(AuAg_24_) shows that this is a lamellar co-crystallization system. All H and C atoms are omitted for clarity. To highlight, the gold atoms in AuAg_24_ and Au_2_Ag_41_ are highlighted in green and pink, respectively.

X-ray crystallographic analysis revealed that AuAg_24_ adopts an icosahedral M_13_ kernel structure, with six, one-dimensional Ag_2_S_3_ motifs surrounding 12 Ag atoms surrounding a central Au atom (Fig. 2ai-ii). The Au_2_Ag_41_ structure comprises a rod-shaped M_25_ kernel composed of two M_13_ structural units (Fig. 2aiii), wrapped by a “cage” like frame composed of the outer Ag-S-P layer (Fig. 2aiv). Detailed structural analyses of both nanoclusters are shown in Supplementary Fig. 2. The bond lengths of AuAg_24_ and Au_2_Ag_41_ were similar—for example, the average Au_kernel_-Ag_kernel_ and Ag_kernel_-Ag_kernel_ bond lengths were 2.91 Å for Au_2_Ag_41_, slightly longer than in AuAg_24_ (2.88 Å). The Au_2_Ag_41_ adopted the same M_25_ kernel as the reported [(p-Tol_3_P)_10_Au_13_Ag_12_Br_8_]^+^^40^, and the average bond lengths of Au_2_Ag_41_ (2.91 Å) were almost identical with that of [(p-Tol_3_P)_10_Au_13_Ag_12_Br_8_]^+^ (2.92 Å) (Supplementary Fig. 3). AuAg_24_ and Au_2_Ag_41_ can be regarded as a structural unit in a stacked three-dimensional structure (Fig. 2b–d). The occupancy of each nanocluster in the unit cell was 50%, and the interlayer distance of lamellar co-crystallization was 24.72 Å (calculated from the gap between each Au plane). The molar ratio of Au_2_Ag_41_ and AuAg_24_ was further confirmed by ^1^H NMR spectroscopy (Supplementary Fig. 4); the integral area ratio of the benzene ring region (–C_6_H_5_) to the partial methylene (–CH_2_) region was 1:6.7, consistent with the theoretically calculated result of 1:6.6. Additionally, X-ray photoelectron spectroscopy (XPS) confirmed the elemental composition of the two nanoclusters (Supplementary Figs. 5–8).

### Dynamic growth process of the bi-nanocluster

**Fig. 3:**
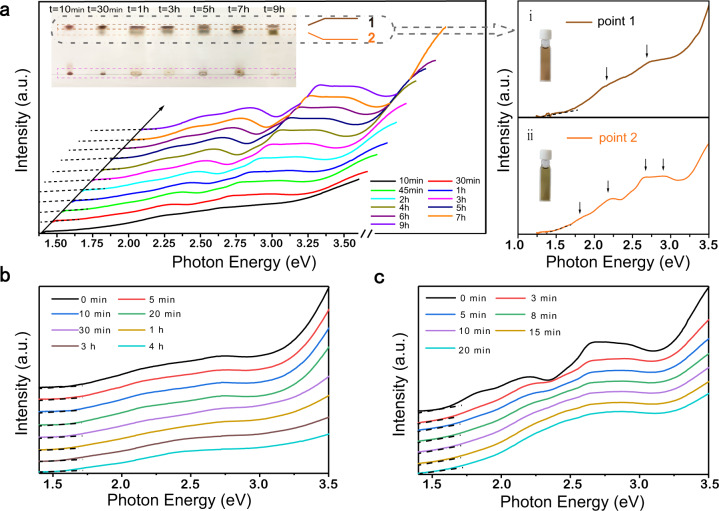
Dynamic growth process of the bi-nanocluster. **a** Time-dependent UV–Vis spectra of the reaction process from 10 min to 9 h plotted on the photon energy scale. The UV–Vis spectra of thin-layer chromatography component points dissolved in dichloromethane (ai) “point 1”, (aii) “point 2”. Insert plots: thin-layer chromatography indicates the separation of reactants at different time interval;digital photos of the dichloromethane solution of separate components. Time-dependent UV–Vis spectra showed the disappearance of totally structured features of the **b** “point 1”, **c** “point 2” at room temperature. “Points 1 and 2” represent AuAg_24_ and Au_2_Ag_41_, respectively.

In order to elucidate the nanocluster formation process, we undertook a thin-layer chromatography study based on the time-dependent absorption peak variation in the UV–Vis spectra (Fig. 3a). “Point 1” appeared on the thin-layer chromatography plate within ten minutes of reaction initiation. As the reaction progressed, the second “point 2” gradually appeared, indicating the formation of a new component. At the same time, we obtained the UV–Vis spectra of two key points plotted on the photon energy scale (Fig. 3ai-ii). Two absorption peaks centered at ~2.17, and 2.68 eV were observed on the spectrum of “point 1”, while four absorption peaks were observed on the spectrum of “point 2” centered at ~1.79, 2.17, 2.68, and 2.90 eV, respectively.

To identify the corresponding clusters, we crystallized component 1 (“point 1”) and component 2 (“point 2”), respectively. The stabilities of components 1 and 2 were tracked by UV–Vis spectra (Fig. 3b, c). The absorption peaks centered at ~2.17, and 2.68 eV corresponding to component 1 disappeared after 4 h, whereas the peaks centered at ~1.79, 2.17, 2.68, and 2.90 eV corresponding to component 2 disappeared within 20 min. Based on this observation, both component 1 and component 2 were concluded to be unstable at room temperature. Accordingly, we added a counterion (tetraphenylphosphonium bromide) to the solution of component 1 and component 2. Single crystal X-ray diffraction (SC-XRD) results showed that component 1 was AuAg_24_ (Supplementary Fig. 9). The structure analysis showed it to be the negatively charged, eight-electron structure [AuAg_24_(SR)_18_]^−^[PPh_4_]^+^ (i.e., 1 (Au 6s^1^) + 24 (Ag 5s^1^) − 18 (SR) − (−1) (charge) = 8). Thus, Au_2_Ag_41_, which has a positive charge, acts as its counter ion in the co-crystal system.

The characteristic absorption peaks of [AuAg_24_(SR)_18_]^−^[PPh_4_]^+^ were centered at 570 nm and 463 nm (2.17 eV and 2.68 eV, respectively)—broader, and blue-shifted compared to published absorption data for AuAg_24_ (2,4-DMBT)_18_ (2,4-DMBT = 2,4-dimethylbenzenethiol) nanocluster— perhaps due to a ligand effect (Supplementary Fig. 10)^41^. Despite extensive experimentation with a variety of counterions, we were unable to stabilize component 2, the instability of which precluded its crystallization. To identify “point 2”, we resorted to comparison of its UV–Vis spectra with that of Au_2_Ag_41_, obtained by subtracting the UV–Vis spectrum of [AuAg_24_(SR)_18_]^−^[PPh_4_]^+^ from the UV–Vis spectrum of (Au_2_Ag_41_)■(AuAg_24_) at an equimolar concentration (Supplementary Fig. 11). Absorption peaks centered at ~443, 472 and 570 nm and 710 nm (corresponding to excitation energies of 2.80, 2.63, 2.17, and 1.75 eV) were observed.

Time-dependent UV–Vis spectra were recorded, to monitor the reaction (Fig. 3a). Characteristic peaks at 2.17 eV and 2.68 eV corresponding to AuAg_24_ appeared within the first 10 min. Later, new peaks centered at 1.79 eV and 2.90 eV appeared, the intensity of which gradually increased. These reflect formation of another component, Au_2_Ag_41_ and the formation of a stable bi-nanocluster system. ^1^H NMR spectra were also acquired, to further track the reaction process and confirm the relationship between the two nanoclusters. At *t* = 10 min of the reaction, no signals corresponding to the benzene ring region could be observed in the ^1^H NMR spectrum, indicating that the Au_2_Ag_41_ stabilized by both thiol and phosphine ligands was not formed. As the reaction continued, the peaks centered at 7.0–8.0 ppm gradually increased in intensity compared with the peak centered at 3.0 ppm ascribed to S-CH of cyclohexanethiol ligand (–CH), indicating the formation of Au_2_Ag_41_ (Supplementary Fig. 12).

### Mechanism of effect between the bi-nanocluster

The above results suggested that AuAg_24_ was produced before Au_2_Ag_41_, but did not inform the relationship between the two. We considered three possibilities: (i) AuAg_24_ induces the formation of Au_2_Ag_41_ in the system; (ii) AuAg_24_ is transformed into Au_2_Ag_41_; and (iii) the two are formed independently.

**Fig. 4:**
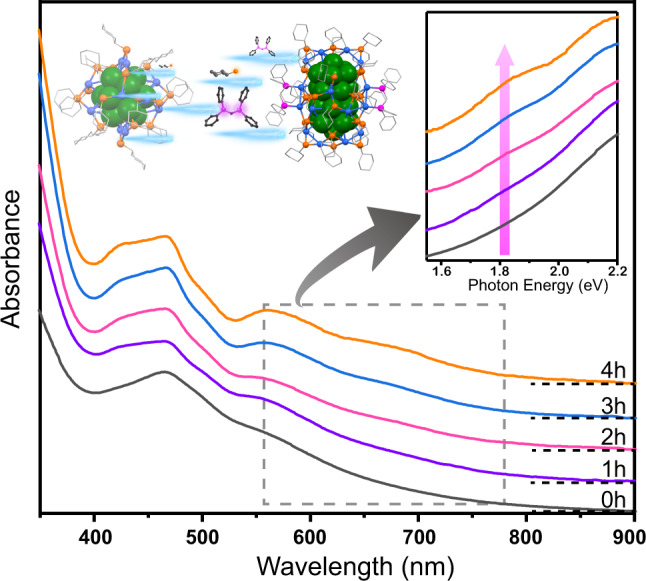
Time-dependent UV–Vis spectra of the nanoclusters. Monodisperse AuAg_24_ is converted to (Au_2_Ag_41_)■(AuAg_24_) bi-nanocluster system in the presence of DPPM. Inset is the amplified photoelectron spectra, which suggest the formation of Au_2_Ag_41_ nanocluster.

To map out the relationship between AuAg_24_ and Au_2_Ag_41_, we designed a conversion reaction. A sample of AuAg_24_ was dissolved in a mixture of dichloromethane and methanol. Then, phosphine and mercaptan ligands were added. The UV–Vis spectra of the mixture were recorded every hour from 0 min to 4 hr (Fig. 4). At 0 min, the UV–Vis spectra exhibited the characteristic absorption peaks of AuAg_24_ at 463 nm and 570 nm. Gradually, new absorption peaks appeared at 695 nm and 425 nm. The spectrum of final product (after 4 h) was identical to that of the bi-nanocluster. These results indicated that the partial conversion of AuAg_24_ to Au_2_Ag_41_ could be induced by the addition of ligands.

**Fig. 5:**
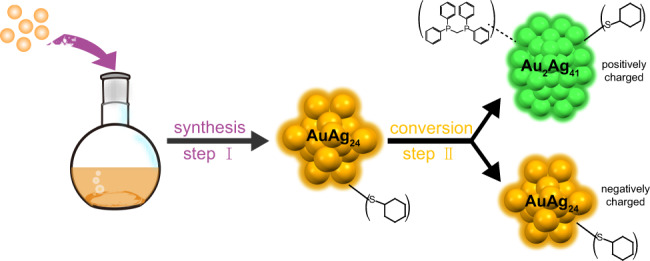
Schematic diagram of the synthesis route for (Au_2_Ag_41_)■(AuAg_24_) bi-nanocluster system. Color code: AuAg_24_: orange; Au_2_Ag_41_: green.

Based on the above results, a mechanism of the transformation and interaction between AuAg_24_ and Au_2_Ag_41_ is proposed (Fig. 5). The reaction is divided into two steps: (I) The negatively charged AuAg_24_ was formed at the beginning of the reaction; (II) Part of the AuAg_24_ nanoclusters were converted to oppositely charged Au_2_Ag_41_ due to the instability of AuAg_24_ in the absence of suitable counterions. Briefly, AuAg_24_ is susceptible to decomposition due to the absence of a suitable counter ion salt protection, causing part of it to be converted into a larger cluster with opposite valence as counter ion. These two types of nanoclusters stabilized each other to form a stable interdependent bi-nanocluster system (Supplementary Fig. 13). The behavior of the conversion from unstable nanoclusters to stable co-crystal system exhibit the synergy between different nanoclusters, which sheds light on the further preparation of new types of nanoparticles in the future.

## Discussion

In summary, we have successfully synthesized a bi-nanocluster system and mapped out the relationship between its two constituent nanoclusters. Owing to the lack of suitable counter ion, the negatively charged AuAg_24_ nanocluster partially converts to the Au_2_Ag_41_ nanocluster, which bears the opposite charge. Thus, the two nanoclusters act as counter ions of each other, establishing a stable and interdependent system. The interdependent effect revealed in this work further advances the understanding of inter-nanocluster correlations.

## Methods

### Materials

Silver nitrate (AgNO_3_, 99.0%), tetrachloroauric(III) acid (HAuCI_4_·3H_2_O, 99.99%, metals basis), tetraphenylphosphonium bromide (PPh_4_Br, 98%), cyclohexyl mercaptan (C_6_H_12_S, 98%), bis-(diphenylphosphino)methane (Dppm, 97%), tetraphenylboron sodium (NaBH_4_, 99%), methanol (CH_3_OH, HPLC, Aldrich), acetone (CH_3_COCH_3,_ Aldrich), dichloromethane (CH_2_Cl_2_, HPLC, Aldrich), methylbenzene (C_6_H_5_CH_3_, Aldrich), ethanol (CH_3_CH_2_OH, Aldrich), acetonitrile (C_2_H_3_N, Aldrich), hexane (C_6_H_14_, Aldrich) were purchased from Sigma-Aldrich. It is worth noting that the reagents used were not further purified.

### Synthesis of the (Au_2_Ag_41_)■(AuAg_24_) co-crystal

AgNO_3_ (60 mg) and bis-(diphenylphosphino)methane (Dppm, 40 mg) were added to a 15 mL methanol solution, and the Ag-Dppm complex was formed after vigorous stirring. Gold salt (HAuCl_4_ • 3H_2_O, 4 mg) was injected into the reaction solution. After continuing to stir for 10 min, cyclohexyl mercaptan (C_6_H_12_S, 100 mg) was added, and the solution changed from white and turbid to yellow and clear. The stirring continued for 20 min until the color of the reaction no longer changed. Then, drop-wise addition of 2 mL of NaBH_4_ ethanol solution (25 mg) to the reaction, the color of the reaction mixture changed to yellow and then to dark. This solution was incubated for 12 h at room temperature. The solution was centrifuged to give the black crude product, which was washed by methanol, then dissolved in toluene to prepare a saturated solution. A certain amount of n-hexane was added thereto and the solution was placed in a refrigerator, and rod-shaped black crystals were obtained in about 7 days.

### Synthesis of AuAg_24_ nanocluster

Typically, we obtained a yellow clear solution according to the above co-crystallization method. Immediately, the drop-wise addition of ice-cold Ethanol NaBH_4_ (20 mg in 2 mL Ethanol). The mixed solution was continuously stirred for 7 h in an ice bath, after which the brown compound was isolated by purification and dissolved in CH_2_Cl_2_. After that, the methanolic PPh_4_Br (30 mg in 2 mL methanol) was added to the solution and which was crystallized in CH_2_Cl_2_/hexane for about 14 days in a refrigerator to obtain black hexagonal crystals.

### Converting AuAg_24_ into Au_2_Ag_41_ nanocluster

Typically, 28 mg of [AuAg_24_(SR)_18_]^−^ [PPh_4_]^+^ nanocluster was dissolved in 15 mL of dichloromethane and methanol 1:1 mixed solution. After that, 30 μL of cyclohexyl mercaptan and 3 mg of bis-(diphenylphosphino)methane were added to the solution, and then approximately 2 mg of NaBH_4_ was added to the solution. Reacted for 4 h at room temperature and oxygen, the final product was collected with a yield of about 26.8%.

### Characterization

Ultraviolet−visible (UV−Vis) absorption spectra were recorded on an UV-6000PC spectrophotometer. X-ray photoelectron spectroscopy (XPS) measurements were performed on Thermo ESCALAB 250 configured with a monochromated Al Kα (1486.8 eV) 150 W X-ray source, 0.5 mm circular spot size, a flood gun to counter charging effects, and the analysis chamber base pressure lower than 1 × 10^−9^ mbar; data were collected with FAT = 20 eV. Nuclear magnetic resonance (NMR) analysis was performed on a Bruker Avance spectrometer operating at 400 MHz for CD_2_Cl_2_ was used as the solvent to dissolve ∼5 mg clusters; the residual solvent peak (i.e.,^1^H at 5.32 ppm) was used as reference. The data collection for single crystal X-ray diffraction was carried out on Stoe Stadivari diffractometer under liquid nitrogen flow at 170 K, using graphite-monochromatized Cu Kα radiation (*λ* = 1.54186 Å). Data reductions and absorption corrections were performed using the SAINT and SADABS programs, respectively.

## Supplementary information

Supplementary Information

## Data Availability

The X-ray crystallographic coordinates for structures reported in this work have been deposited at the Cambridge Crystallographic Data Center (CCDC), under deposition numbers CCDC-2007161 and 2007612. These data can be obtained free of charge from the Cambridge Crystallographic Data Centre via www.ccdc.cam.ac.uk/data_request/cif, which has been mentioned in the article.
