# Supplementary material for: Interdependence between nanoclusters AuAg24 and Au2Ag41
Source: Nat Commun. 2021 Feb 3;12:778. doi: 10.1038/s41467-021-21131-5 (PMC7858706; doi:10.1038/s41467-021-21131-5)
Supplement: Supplementary file 1 — Supplementary Information [file 41467_2021_21131_MOESM1_ESM.pdf]

Supplementary Information

Interdependence Between Nanoclusters $AuAg_{24}$ and Au_2Ag_{41}

Danyu Liu, Wenjun Du, Shuang Chen, Xi Kang, Along Chen, Yaru Zhen, Shan Jin, Daqiao Hu*, Shuxin Wang* & Manzhou Zhu*

*Correspondence to: zmz@ahu.edu.cn

Contents **Pages**

Supplementary figures 1-13 S2-S8

Supplementary tables 1-2 S9-S10

Supplementary References S11

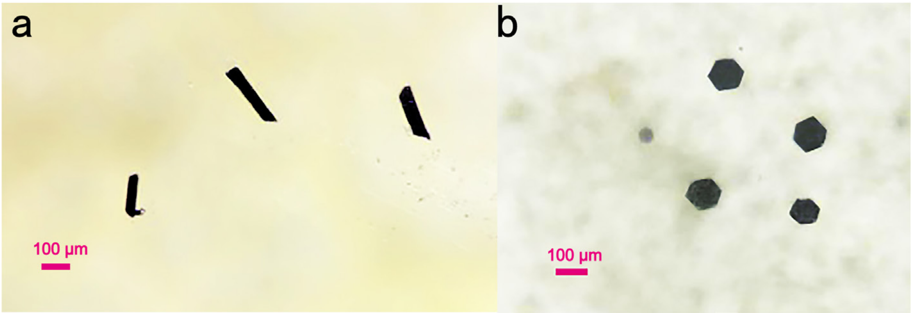

Supplementary Fig. 1 Crystals of the a) (Au_2Ag_{41})▪$(AuAg_{24})$ co-crystal and b) $[AuAg_{24}(SR)_{18}]^-[PPh_4]^+$ captured under a microscope.

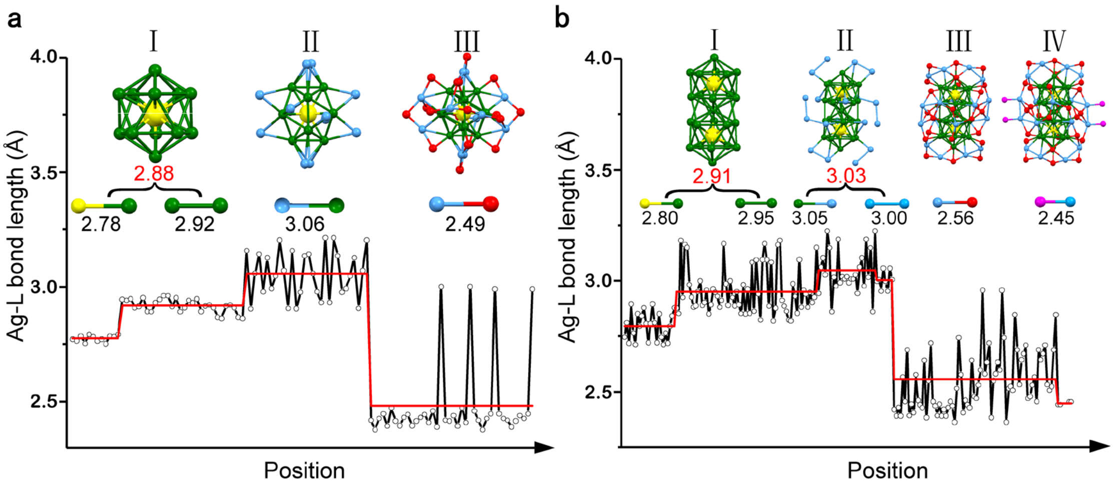

Supplementary Fig. 2 Ag-L bond lengths of the (a) $AuAg_{24}$; (b) Au_2Ag_{41}. Wherein L = Au, Ag, S, P elements. Color label: yellow = gold; green, sky blue = silver; red = sulfur; fuchsia = phosphine.

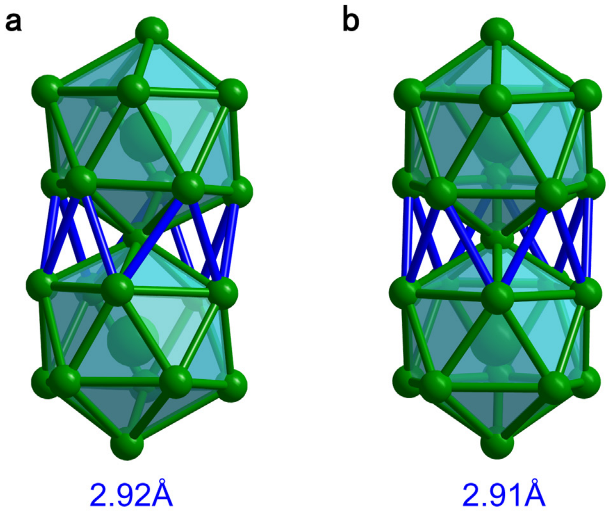

Supplementary Fig. 3 The Au/Ag-Ag bond length of kernel of a) $[(p\text{-}Tol_3P)_{10}Au_{13}Ag_{12}Br_8]^+$; b) Au_2Ag_{41}. Color label: Green = gold or silver.[1]

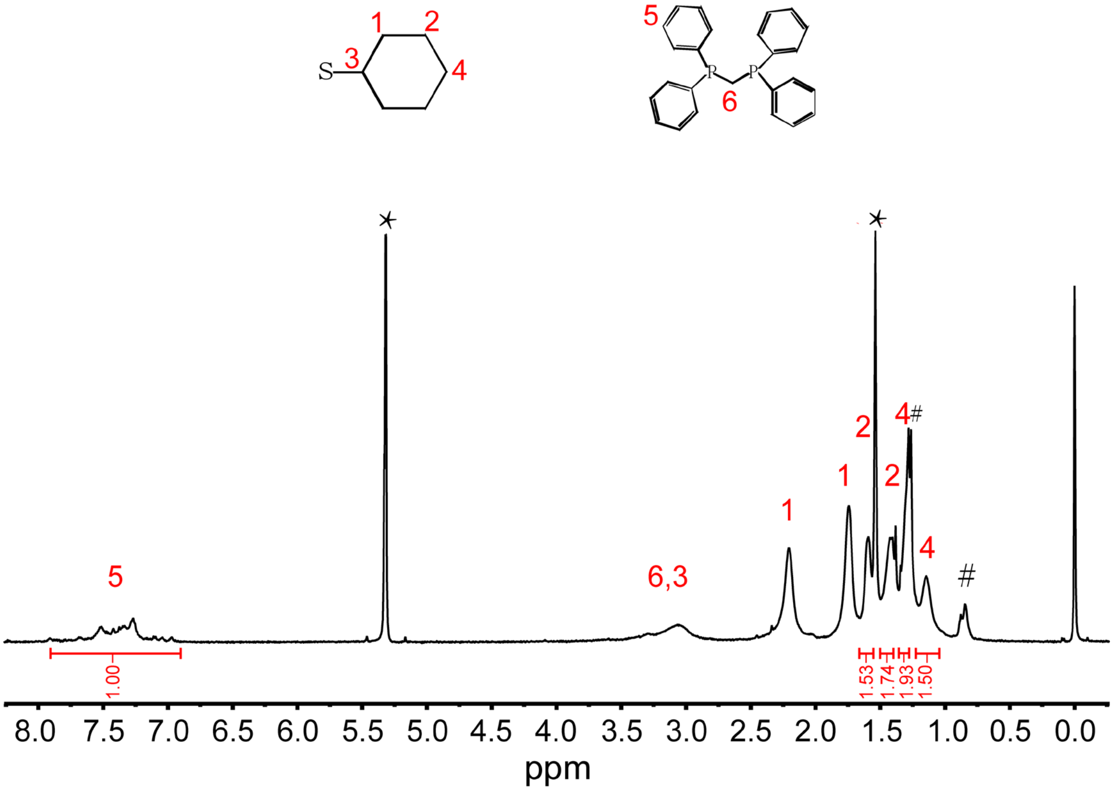

Supplementary Fig. 4 ^{1}H NMR spectrum of the (Au_2Ag_{41})▪($AuAg_{24}$) co-crystal in CD_2Cl_2. The peak labeled * is from the solvent and the peak labeled # is from n-hexane with a chemical shift of 0.81 ppm and 1.23 ppm. (The ligand distribution is H in the vicinity of the benzene ring region, and the number is 5, and the cyclohexyl group in the cyclohexyl mercaptan has four environments of H, and columns 1-4. The last 6 represents the -CH_2 group in the phosphine ligand).

$AuAg_{24}(SC_6H_{11})_{18}$ X

$Au_2Ag_{41}(SC_6H_{11})_{26}(Dppm)_2$ 1-X

$$\frac{1}{1.53+1.74+1.93+1.50}=\frac{20\times 2(1-X)}{3\times 2\times 18X+3\times 2\times 26(1-X)}$$

$$\frac{1}{6.70}=\frac{40-40X}{156-48X}$$

$$X = 0.509$$

So the molar ratio of $AuAg_{24}$ to Au_2Ag_{41} is approximately 1.

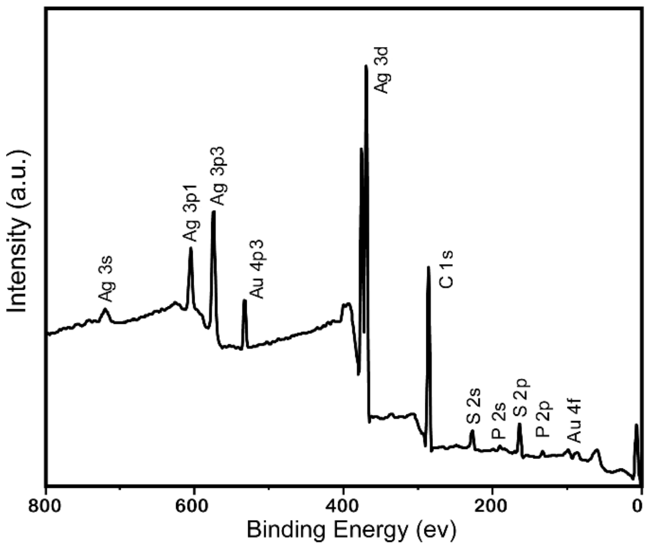

Supplementary Fig. 5 XPS spectrum of the (Au_2Ag_{41})▪$(AuAg_{24})$ co-crystal.

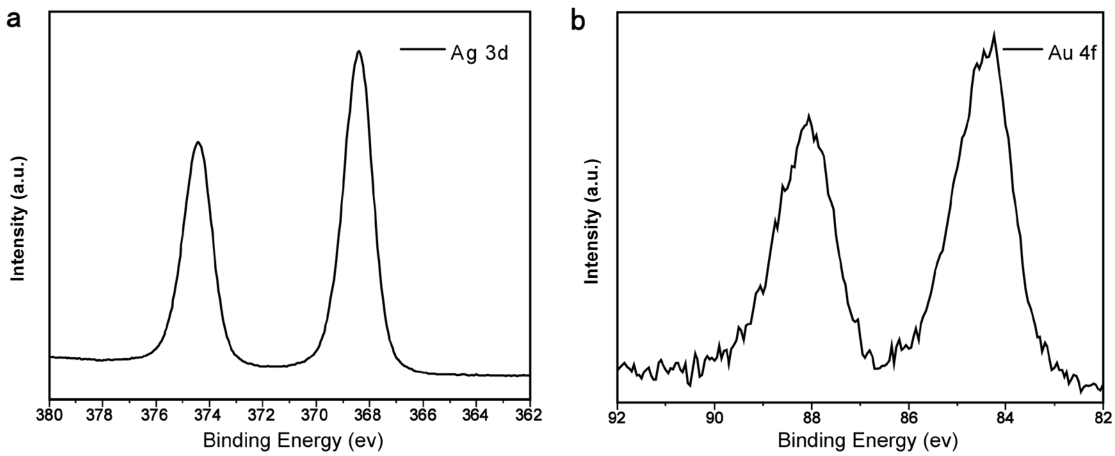

Supplementary Fig. 6 XPS spectra of a) Ag_{3d} b) Au_{4f} in the (Au_2Ag_{41})▪$(AuAg_{24})$ co-crystal.

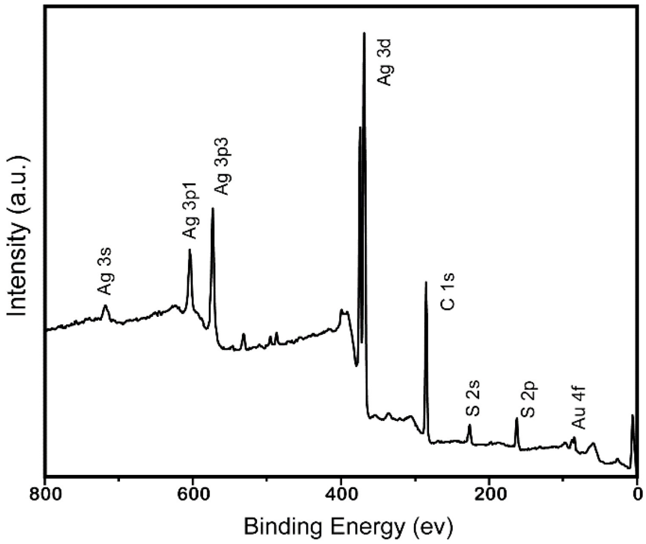

Supplementary Fig. 7 XPS spectrum of $[AuAg_{24}(SR)_{18}]^{-}[PPh_4]^{+}$ nanocluster.

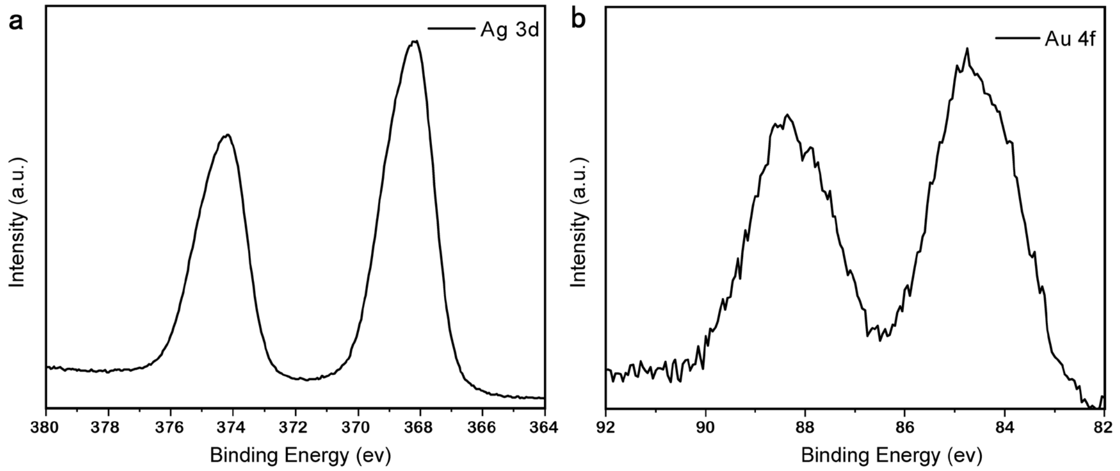

Supplementary Fig. 8 XPS spectra of a) Ag_{3d} b) Au_{4f} in the $[AuAg_{24}(SR)_{18}]^{-}[PPh_4]^{+}$ nanocluster.

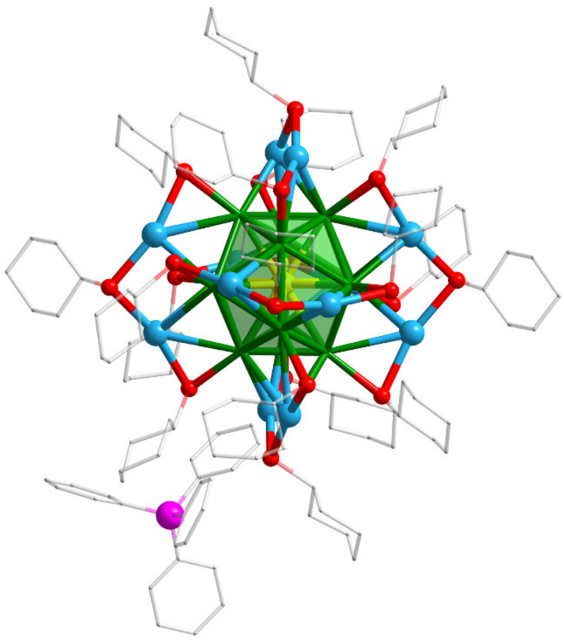

Supplementary Fig. 9 Crystal structure of the $[AuAg_{24}(SR)_{18}]^{-}$ $[PPh_4]^{+}$ nanocluster. Color labels: yellow, Au; green or sky blue, Ag; red, S; magenta, P; gray, C. All H atoms are omitted for clarity.

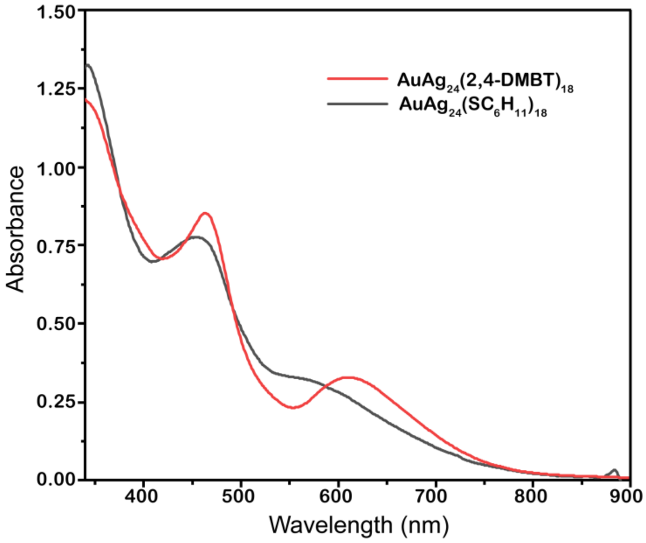

Supplementary Fig. 10 The UV-vis absorption spectra of the $AuAg_{24}$ $(SC_6H_{11})_{18}$ nanocluster and the $AuAg_{24}$ (2,4-DMBT)$_{18}$ nanocluster.

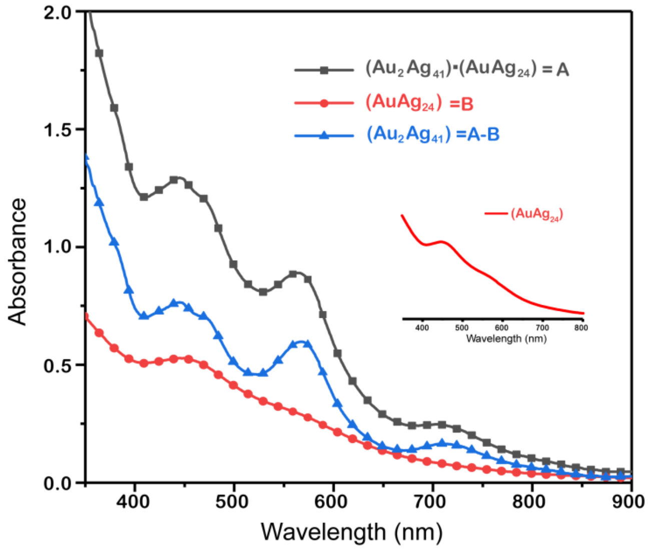

Supplementary Fig. 11 The UV-vis absorption spectra of (Au_2Ag_{41})▪$(AuAg_{24})$ co-crystal and purified $[AuAg_{24}(SR)_{18}]^-$ $[PPh_4]^+$ nanocluster dissolved in dichloromethane.

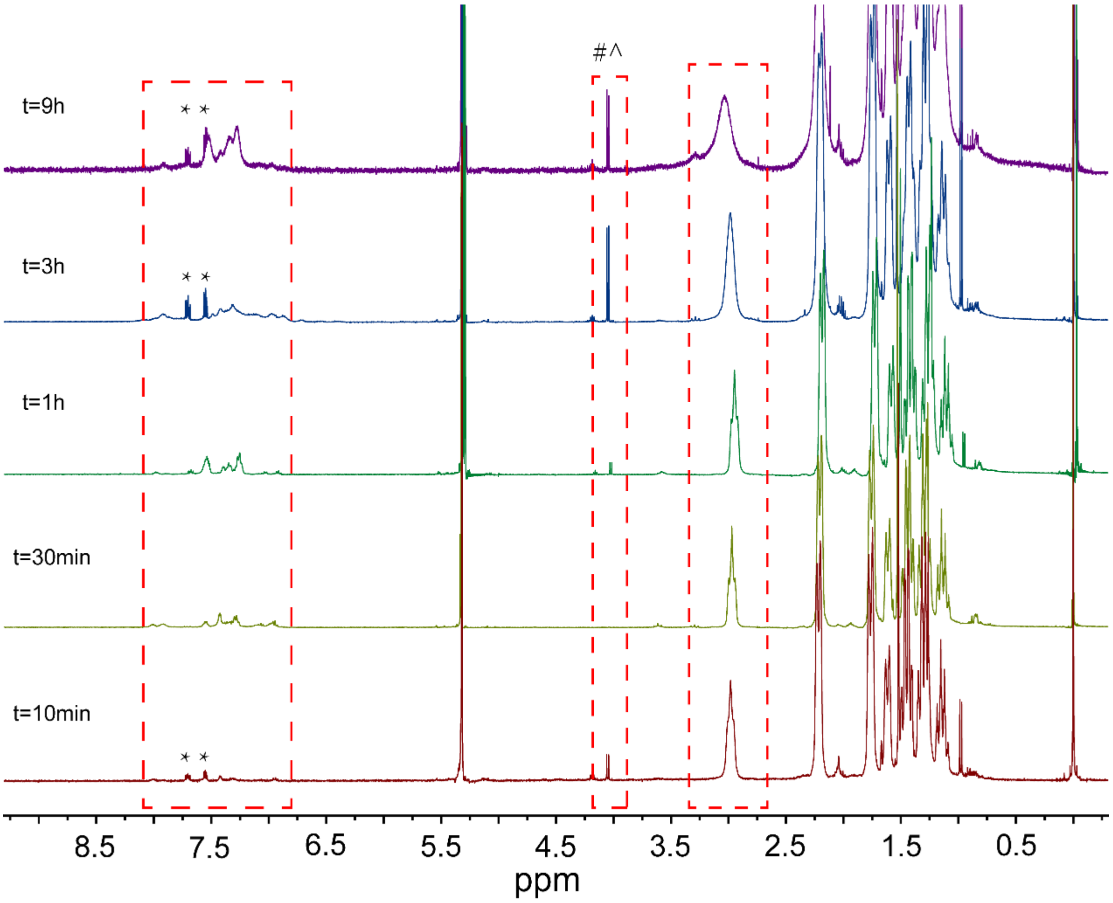

Supplementary Fig. 12 ^{1}H NMR spectra of the reaction process in CD_2Cl_2 measured at 10min-9h periods. Experimental details: After reduction by $NaBH_4$, the reaction solution was removed after vigorous stirring. The excess phosphine ligand and the thiol ligand were removed by repeated washing with an acetonitrile solution. After the treated solid powder was dried, ~5 mg was weighed and dissolved into 600 μl of CD_2Cl_2, and finally the solution was transferred to a nuclear magnetic tube and the spectrum after the reaction was recorded for corresponding time. Where (*) belongs to the residual phosphine ligand Dppm peaks, and the 5.32 ppm peak is labeled as CH_2Cl_2. The chemical shift at 2.19 ppm is the peak of the residual solvent acetonitrile. (#) indicates a resonance peak derived from ethanol, and (^) may be assigned to a resonance peak of methanol.

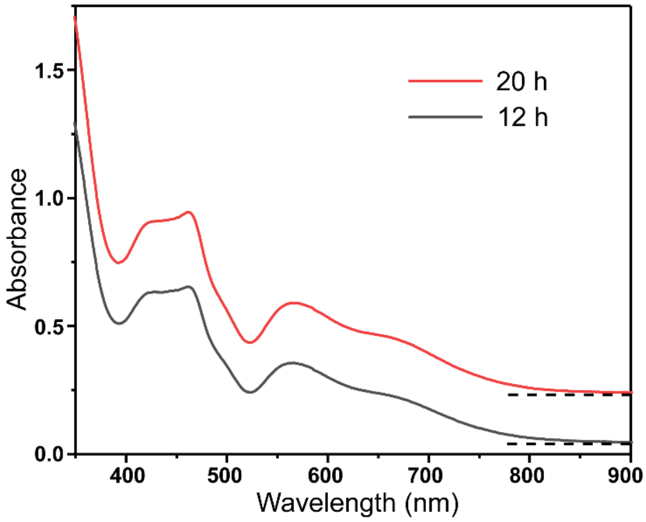

Supplementary Fig. 13 The UV-vis absorption spectra of (Au_2Ag_{41})▪($AuAg_{24}$) bi-nanocluster solution after reacting for 12 (black line) and 20 hours (red line).

Supplementary Table 1 Co-crystal data and structure refinement for $AuAg_{24}(C_6H_{11}S)_{18}$ and $Au_2Ag_{41}(C_6H_{11}S)_{26}(Dppm)_2$

Identification code	$AuAg_{24}$ and Au_2Ag_{41}
Empirical formula	$C_{314}H_{528}Ag_{65}Au_3P_4S_{44}$
Formula weight	13440.30
Temperature/K	120
Crystal system	triclinic
Space group	P-1
a/Å	20.335(3)
b/Å	23.601(6)
c/Å	24.721(6)
α /°	77.785(19)
β /°	81.161(16)
γ /°	87.434(16)
Volume/Å^3	11457(4)
Z	1
ρ_{calc} g/cm^3	1.948
μ /mm^{-1}	27.717
F(000)	6468
Radiation	CuKa (λ = 1.54186)
2Θ range for data collection/°	3.698 to 134.514
Reflections collected	39814
Independent reflections	39814 [R_{int} = 0.0622, R_{sigma} = 0.0557]
Data/restraints/parameters	39814/0/1891
Goodness-of-fit on F^2	1.073
Final R indexes [I>=2σ (I)]	R_1 = 0.0652, wR_2 = 0.1835
Final R indexes [all data]	R_1 = 0.0842, wR_2 = 0.1955
Largest diff. peak/hole / e Å^{-3}	10.23/-2.33

Supplementary Table 2 Crystal data and structure refinement for $AuAg_{24}(C_6H_{11}S)_{18}$

Identification code	$AuAg_{24}$
Empirical formula	$C_{132}H_{218}Ag_{24}AuPS_{18}$
Formula weight	5198.95
Temperature/K	120
Crystal system	triclinic
Space group	P-1
a/Å	17.279
b/Å	18.944
c/Å	19.125
α /°	59.99
β /°	66.54
γ /°	65.98
Volume/Å^3	4799.6
Z	1
ρ_{calc} g/cm^3	1.799
μ /mm^{-1}	22.691
F(000)	2520.0
Radiation	CuKa (λ = 1.54186)
2Θ range for data collection/°	9.296 to 133.986
Index ranges	-20≤h≤17, -22≤k≤22, -22≤l≤17
Reflections collected	36013
Independent reflections	16544 [R_{int} = 0.0479, R_{sigma} = 0.0535]
Data/restraints/parameters	16544/2025/958
Goodness-of-fit on F^2	1.020
Final R indexes [I>=2σ (I)]	R_1 = 0.0804, wR_2 = 0.2287
Final R indexes [all data]	R_1 = 0.1097, wR_2 = 0.2476
Largest diff. peak/hole / e Å^{-3}	3.82/-1.10

Supplementary References

1. Teo, B. K.; Zhang, H. & Shi, X. Molecular architecture of a novel vertex-sharing biicosahedral cluster $[(p\text{-}Tol_3P)_{10}Au_{13}Ag_{12}Br_8](PF_6)$ containing a staggered-staggered-staggered configuration for the 25-atom metal framework. *Inorg. Chem.* **29,** 2083–2091 (1990)
